# Median regression spline modeling of longitudinal FEV_1_ measurements in cystic fibrosis (CF) and chronic obstructive pulmonary disease (COPD) patients

**DOI:** 10.1371/journal.pone.0190061

**Published:** 2017-12-20

**Authors:** Douglas J. Conrad, Barbara A. Bailey, Jon A. Hardie, Per S. Bakke, Tomas M. L. Eagan, Bernt B. Aarli

**Affiliations:** 1 Department of Medicine, University of California, San Diego, United States of America; 2 Department of Mathematics and Statistics, San Diego State University, San Diego, United States of America; 3 Department of Clinical Science, University of Bergen, Bergen, Norway; 4 Department of Thoracic Medicine, Haukeland University Hospital, Bergen, Norway; University of Pittsburgh, UNITED STATES

## Abstract

**Rationale:**

Clinical phenotyping, therapeutic investigations as well as genomic, airway secretion metabolomic and metagenomic investigations can benefit from robust, nonlinear modeling of FEV_1_ in *individual* subjects. We demonstrate the utility of measuring FEV_1_ dynamics in representative cystic fibrosis (CF) and chronic obstructive pulmonary disease (COPD) populations.

**Methods:**

Individual FEV_1_ data from CF and COPD subjects were modeled by estimating median regression splines and their predicted first and second derivatives. Classes were created from variables that capture the dynamics of these curves in both cohorts.

**Results:**

Nine FEV_1_ dynamic variables were identified from the splines and their predicted derivatives in individuals with CF (n = 177) and COPD (n = 374). Three FEV_1_ dynamic classes (i.e. stable, intermediate and hypervariable) were generated and described using these variables from both cohorts. In the CF cohort, the FEV_1_ hypervariable class (HV) was associated with a clinically unstable, female-dominated phenotypes while stable FEV_1_ class (S) individuals were highly associated with the male-dominated milder clinical phenotype. In the COPD cohort, associations were found between the FEV_1_ dynamic classes, the COPD GOLD grades, with exacerbation frequency and symptoms.

**Conclusion:**

Nonlinear modeling of FEV_1_ with splines provides new insights and is useful in characterizing CF and COPD clinical phenotypes.

## Introduction

Longitudinal lung function assessments in Cystic Fibrosis (CF) and Chronic Obstructive Pulmonary Disease (COPD) are critical for monitoring disease progression and response to therapy. Population-based modeling of lung function typically uses least-squares trendlines, median quantile linear regression, or smoothing spline curves [1–9]. These approaches are useful in assessing long-term disease progression as well as genomic/genetic risk in both CF and COPD populations. However, some studies including airway secretion microbiome and metabolomic studies require quantification of *individual* short-term (i.e. weeks to months) lung function variability. In most cases, individual lung function data is modeled with least-squares trendlines focusing on the slopes and intercepts [5,10]. At least two investigations focused on individual variability as a marker of disease risk in CF and COPD populations [10,11]. However, these studies used one or two variables and only captured some of the full dynamic complexity of a specific individual’s lung function.

The purpose of this analysis was to model lung function dynamics of individuals with median regression splines in order to capture clinically relevant, subject-specific variability. Furthermore we aimed to determine if this variability provides additional insights to CF and established COPD phenotypes as well as commonly assessed clinical parameter.

## Methods

### Study populations

The study was reviewed and approved by the UCSD HRPP (application #081500) and the Norway Regional Ethics Committee (REK 165.08) and performed in accordance with the Declaration of Helsinki and the Good Clinical Practice guidelines. All subjects signed informed consent.

The CF population consisted of all adult (≥18 years of age) patients seen at the San Diego adult CF program at least once during the calendar year 2014 (n = 177). For each CF patient, we collected basic demographic (gender, age), nutritional data (height and weight) and all available clinically-indicated spirometry results dating back to 1/1/2012. Spirometry was performed in accordance with ATS guidelines and normalized using the Hankinson equations [12]. The age*FEV_1_% predicted (AF) product is a derived variable calculated by multiplying the age of the individual at the most recent FEV_1_ by the best FEV_1_% during the previous year and is used to assess disease risk [5,13]. Each CF subject was mapped into a specific CF multi-dimensional clinical phenotype (MDCP) based on the most recent clinical data and the published random forests prediction model [13]. These clinical phenotypes were generated using age, gender, FEV_1_, FVC, height and weight and two derived variables: Body Mass Index (BMI) and the AF product (S1 Table). These phenotypes represent clinically relevant groups that differ not only in the class forming variables but also in microbiology (13).

The COPD data derives from the Bergen Cohort COPD Study (BCCS) [14,15]. Only patients with a minimum of four visits were included in the present study (n = 374). BCCS evaluations were attempted every 6 months for 3 years with an additional visit 3 months after baseline, totaling 8 visits. Smoking habits and exacerbations since last visit were recorded by the study physician at each visit. Breathlessness was quantified using modified Medical Research Council dyspnea scale score (MMRC) [16]. Lung function was normalized to the ECCS 1993 equations [17]. The COPD subjects were classified into their specific GOLD grades of airway obstruction using their FEV_1_% values.

### Nonlinear modeling of Forced expiratory volume 1 (FEV_1_)

We modeled each subject’s FEV_1_% using the estimated median regression spline. A median spline is similar to the traditional cubic smoothing spline except that the conditional median is estimated. The estimation of this spline is reproducible, and generates statistically robust smoothing curves. The median quantile regression spline is not sensitive to technical or biological outlier values [18]. Details of fitting the median splines are found in the Supporting Information (S1 Fig).

For each subject, the time between the first and most recent spirometry was divided into 1000 equally spaced time points. The predicted spline values as well as the estimated first and second derivatives were determined at each time point. From these, the following nine summary variables were determined for each subject: a) the effective degrees of freedom of the median spline, b) the range between the 95^th^ and 5^th^ percentile of the median spline and the derivatives, c) the number of local minima and maxima of the median spline and the derivatives, d) and the median values of the first and second derivative of the splines. The effective degrees-of-freedom is a proxy for the approximate degree of the equivalent polynomial fit needed to generate the median spline and is thus a measure of the variability of the data. Similarly, the number of local maxima and minima identify the instances when the spline or derivatives change direction; another but distinct measure of variability. The range between the 5^th^ and 95^th^ percentile values of the spline and derivatives quantifies the magnitude of change occurring in these curves during the study period. Finally, the median values of the first and second derivatives identify the overall rate of change of lung function and the general curvature of the median spline, respectively (S1 Fig). In addition to these variables, the slopes of the least-squares trendlines and the median of differences from the best FEV_1_% were estimated for each patient [10].

### FEV_1_ dynamic class formation

Class formation used the algorithms previously described [13]. The CF and COPD cohorts were analyzed separately. The nine variables were used to generate a proximity matrix using the unsupervised Random Forests algorithm [13,19]. Partitioning Around Medoids (PAM) clustering was used to create the classes (i.e. k = 3, 4 or 5) [20]. The classification error rates (i.e. out of bag error rates) were minimized using the three-class strategy (i.e. k = 3).

Dimension reduction demonstrated that the use of four variables (the effective degrees of freedom and the ranges between the 5^th^ and 95^th^ percentile of the median regression spline and the two derivatives) created classes with out-of-bag error rates of about 8%.

### Statistical analysis

Notch, mosaic and matrix plots were generated using R Version 3.4.0 (base and vcd packages)[21–23].

## Results

### Patient population

**Fig 1:**
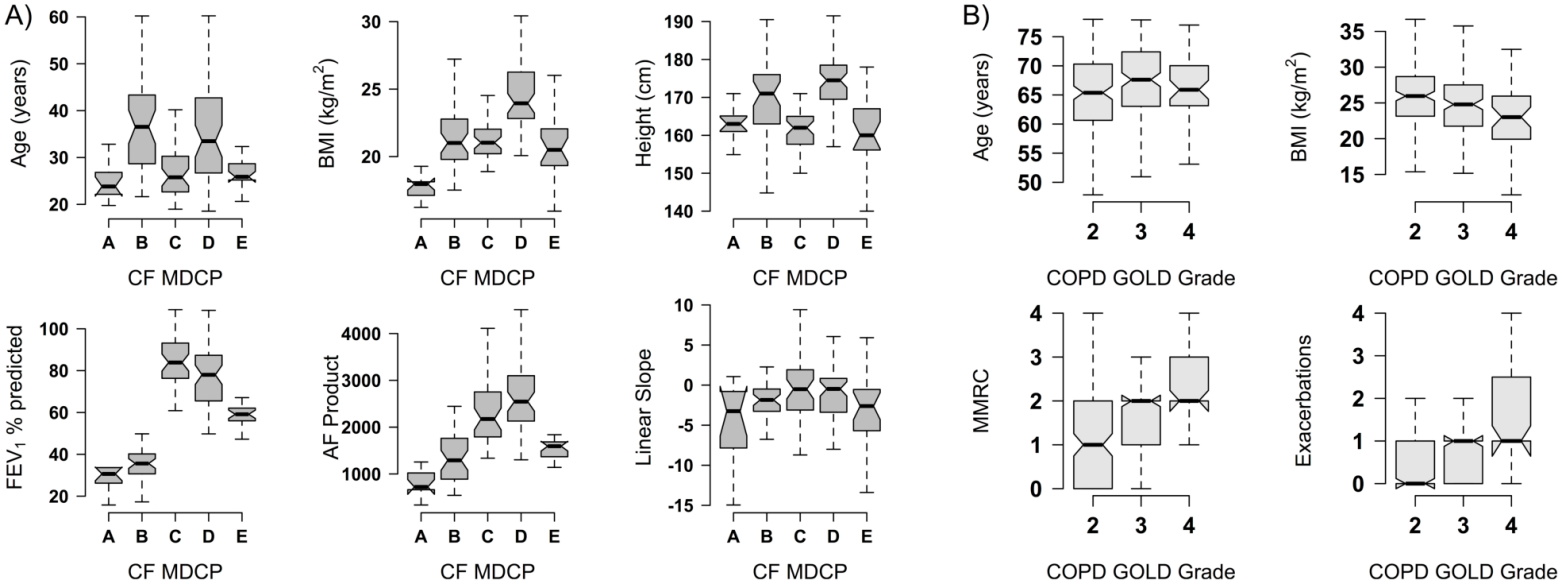
Clinical phenotype characterization. Notched boxplots of basic clinical and physiological characteristics of the CF (Fig 1A) and COPD (Fig 1B) cohorts. Each subject was mapped to either a MDCP class [13] or COPD GOLD grade. The AF product of an individual is the age at the most recent FEV_1_ multiplied by the best FEV_1_% during the study period. The linear slope is the slope of the least squares trendline fitted to the FEV_1_ data during the study period.

For this study, 177 adult CF patients signed consent and had complete datasets including a minimum of 4 spirometry studies (Table 1). This patient population did not differ significantly from other adult CF populations in terms of age, gender, nutritional parameters or lung physiology [13,24]. There was a median of six spirometric assessments per patient in this cohort. Each CF subject was mapped to one of five regional clinical phenotypes and as a group they closely resemble the original published phenotypes (Fig 1A) [13]. The distribution of demographic and physiological parameters between the GOLD grades for the Bergen Cohort COPD Study is shown in Fig 1B [14,15]. There were no clinically significant differences in age, gender, BMI or loss of lung function as assessed by the slope of the least-squares trendlines between the GOLD grades. Patients in GOLD 3 and 4 had higher MMRC scores.

**Table 1:** Baseline demographics in the San Diego CF cohort and the Bergen Cohort COPD study.

	San Diego CF cohort	Bergen Cohort COPD Study
**Subjects (n)**	177	374
**Age (years)**	29 (11)	63 (7)
**BMI kg/m**^**2**^	22 (3)	26 (6)
**Pack-years (years)**	-	40 (23)
**MMRC**	-	1.6
Exacerbation rate*	-	1.4
**FEV**_**1**_ **(%) predicted**	63 (21)†	52 (15)
**FVC (%) predicted**	81 (21)†	93 (18)

Data presented as mean or mean (standard deviation). BMI: Body Mass Index. Pack-years: packs of 20 cigarettes smoked per day x years as a smoker, MMRC: modified Medical Research Council dyspnea scale score–mean. FEV_1_: Forced expiratory volume in 1 second, FVC: Forced Vital Capacity.

*Exacerbation rate the first year of the study.

^†^Best FEV_1_ and FVC (%) predicted for the prior 12 months.

**Fig 2:**
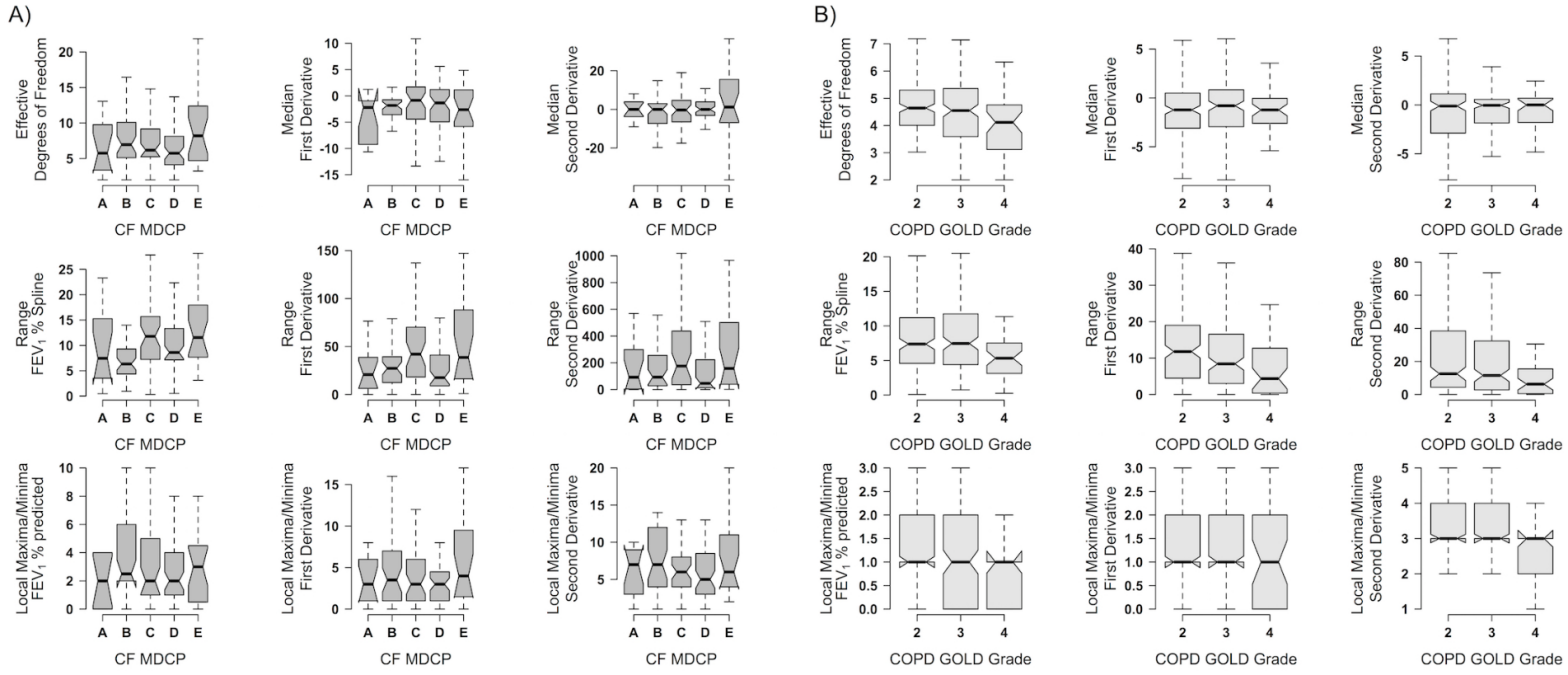
FEV_1_% dynamic variables in CF and COPD clinical phenotypes. Notched boxplots of the FEV_1_% dynamic variables for CF (A) and COPD (B) subjects are shown for each CF MDCP class and COPD GOLD grade.

Lung function dynamic variables in CF MDCP classes and in the COPD GOLD grades. In the CF population, there were no differences in the dynamic variables including the slope of the least-squares trendlines between subjects mapped into the published regional clinical phenotypes except for minor differences in the ranges of the median regression splines and their estimated first derivatives (Fig 2A). Similarly, the BCCS cohort demonstrated slightly smaller ranges of the splines and estimated first derivatives in GOLD grade 4 compared to grade 2. There were no significant differences in the other dynamic variables between the GOLD grades 2–4 (Fig 2B).

### FEV_1_ dynamic class formation

These modest associations of the individual FEV_1_ dynamic variables with the CF clinical phenotypes and COPD GOLD grades suggested that combinations of the variables were needed to characterize the dynamics of the median regression splines. In the CF cohort, the classification error rates using the k = 3 strategy decreased slightly from 10% to 8% with the use of just four of the original nine variables i.e. effective degrees of freedom, and the ranges between the 5^th^ and 95^th^ percentile values of the spline, the first and second derivatives. Similar results were obtained with the COPD cohort data. In both cohorts, the classification error rates were the lowest with the k = 3 and increased with the k = 4,5 or 6 strategies.

**Fig 3:**
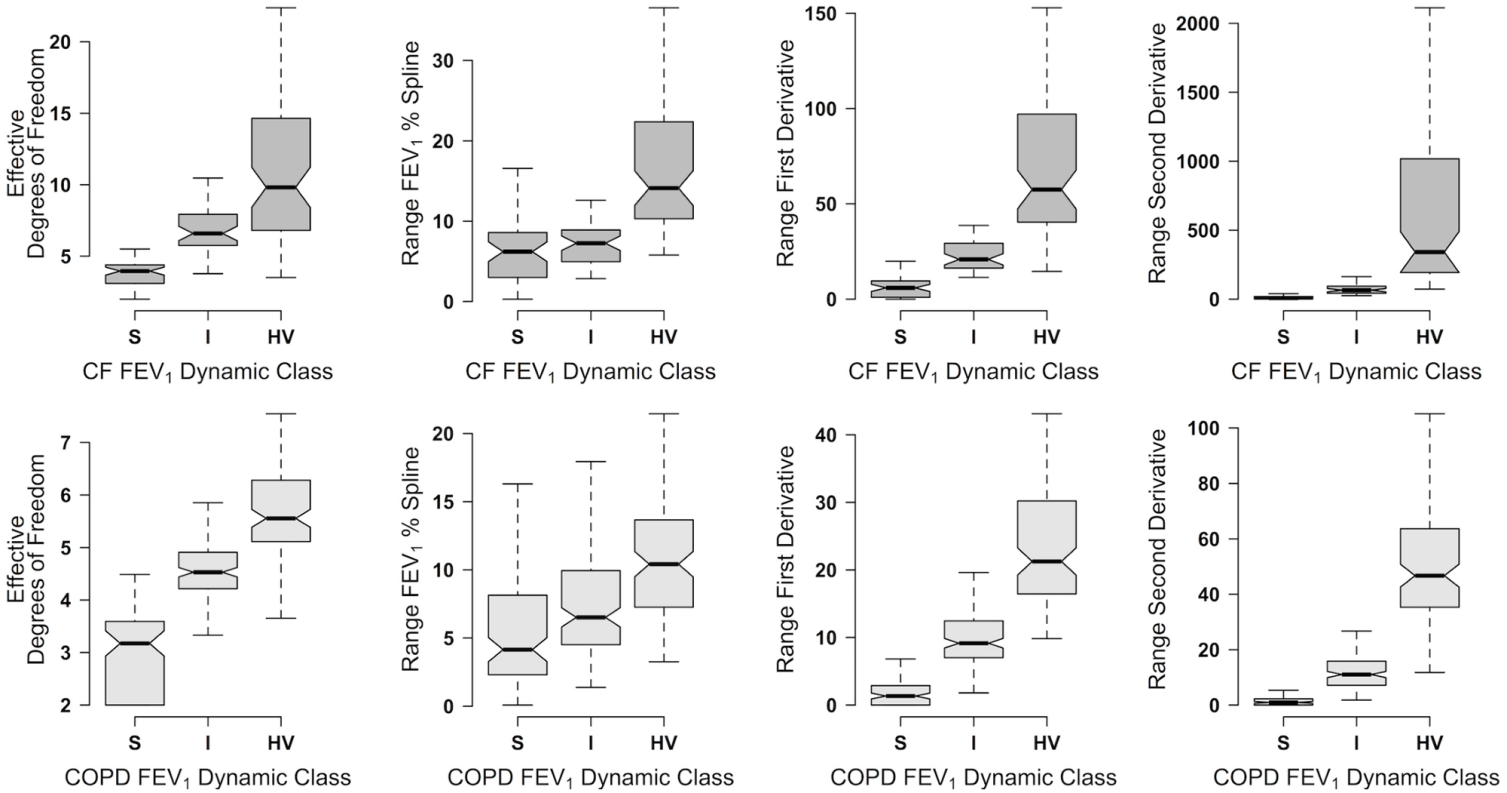
CF and COPD FEV_1_% dynamic classes classifying variables. Notched boxplots demonstrate the distribution of FEV_1_% dynamic classifying variables for CF and COPD subjects in each of the FEV_1_% Dynamic Classes, i.e. stable (S), intermediate (I) and hypervariable (HV).

The three resulting classes or lung function phenotypes differed significantly in terms of the ranges of the splines and the derivatives as well as the effective degrees of freedom. The groups with the highest and lowest ranges and effective degree of freedom were labeled as hypervariable (HV) and stable (S) respectively. The class with values that were between was labeled intermediate (I). The four classifying variables were clearly separated between the CF and COPD classes (Fig 3).

**Fig 4:**
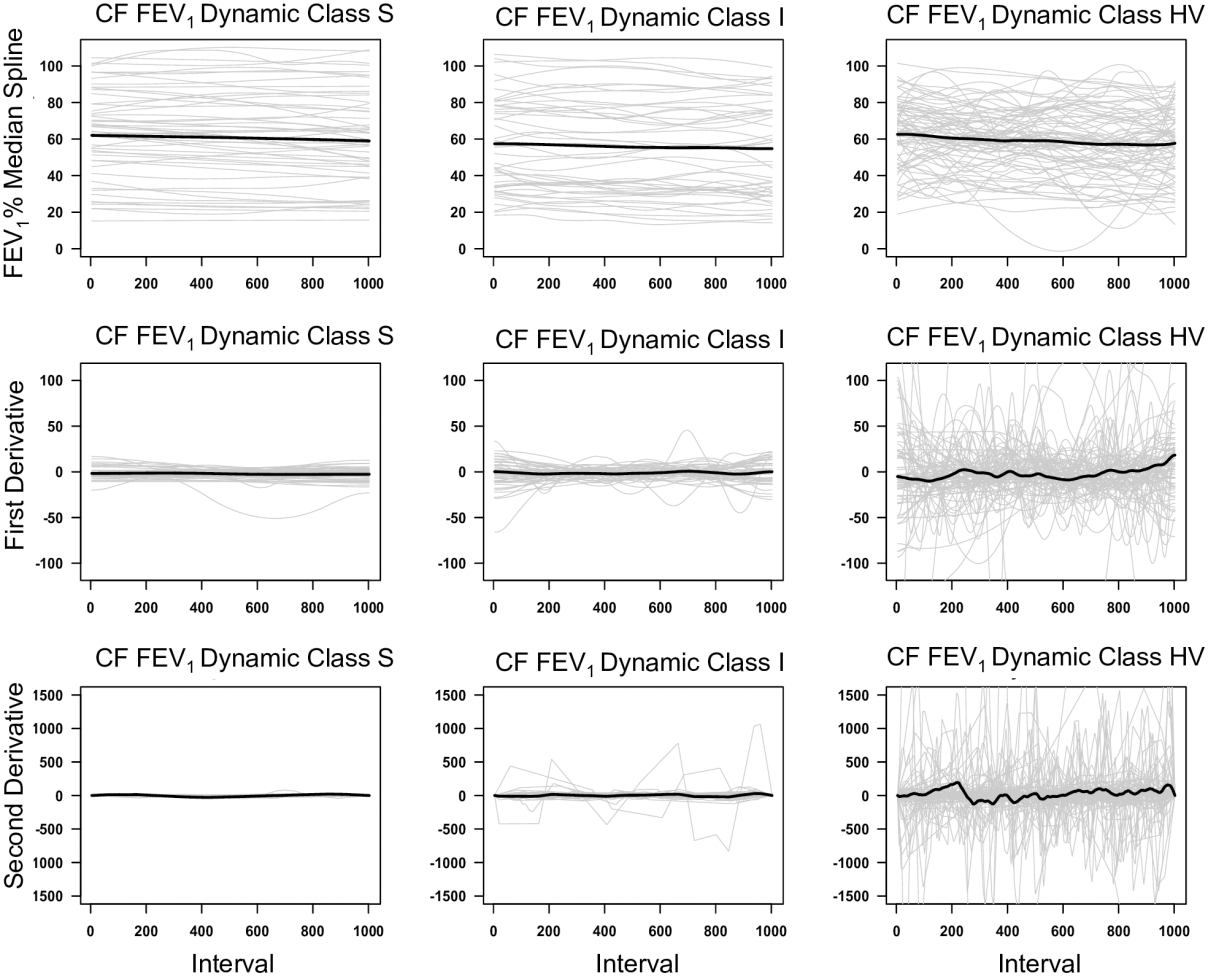
Spaghetti plots of the regional CF cohort. The individual estimated median regression splines and their predicted first and second derivatives are depicted in the three CF FEV_1_% Dynamic Classes. A thousand equally spaced values of the estimated median regression spline and their predicted first and second derivatives were plotted for each subject in each FEV_1_% dynamic class (thin grey lines). The mean value at each interval point is plotted as the black solid line.

Spaghetti plots of the CF subjects show the values of the median spline and the first two derivatives as a function of time (Fig 4). These plots demonstrated that all three classes of splines had similar absolute values and general rate of decline in FEV_1_% predicted. In contrast, the derivative plots clearly demonstrate that the FEV_1_ dynamic class S has little dynamic change whereas class HV was hypervariable. Class I had intermediate variability.

**Fig 5:**
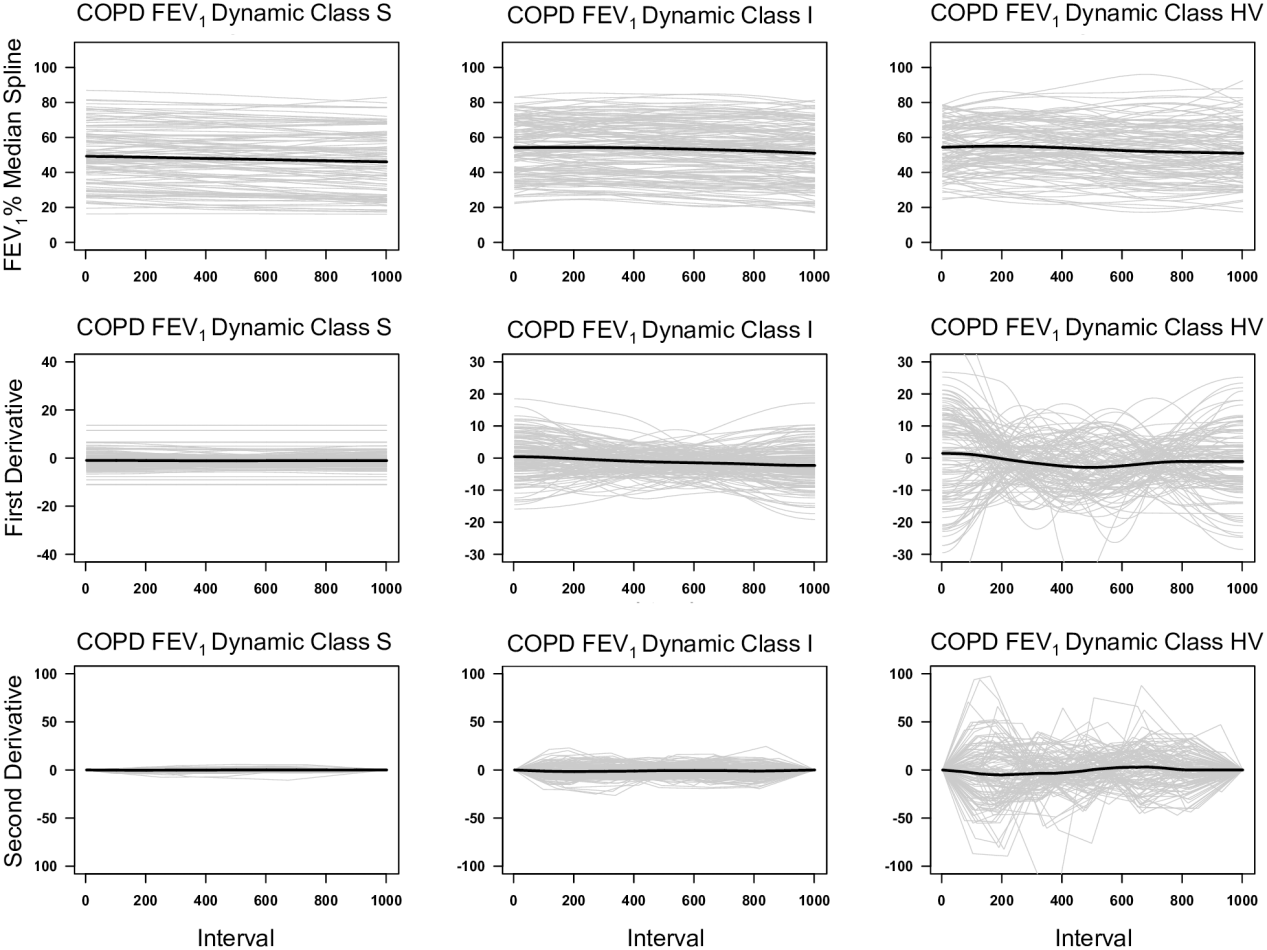
Spaghetti plots of the BCCS COPD cohort. This figure was similarly labeled as Fig 4.

Spaghetti plots of the three COPD FEV_1_ dynamic classes showed similar median spline values and the mean rates of lung function loss in each of the GOLD grades (Fig 5). As with the CF cohort, the plots of the derivatives clearly showed that the COPD FEV_1_ dynamic class S was very stable compared to class HV. Again, class I demonstrated intermediate variability.

### FEV_1_ dynamic classes versus clinical parameters

**Fig 6:**
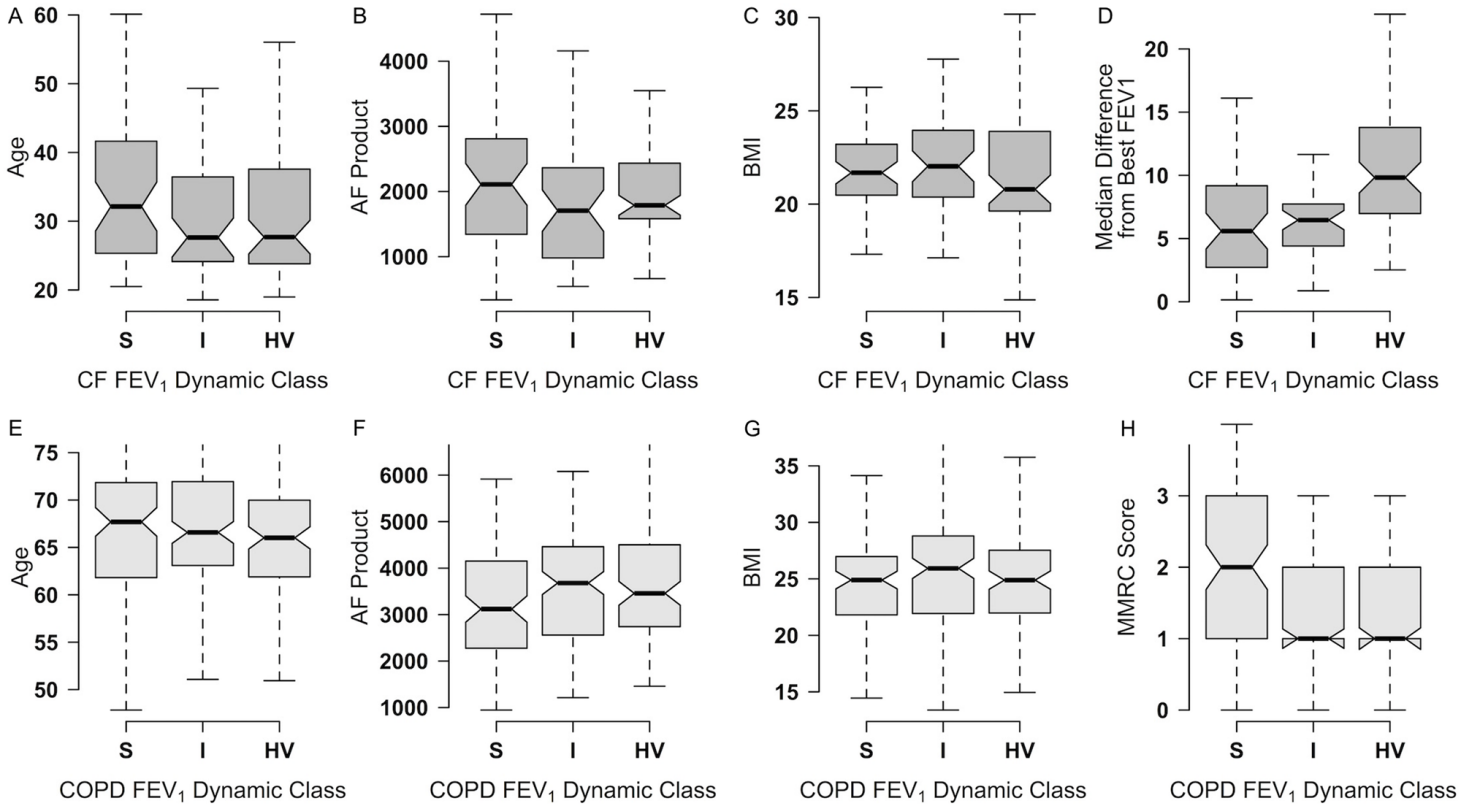
Distribution of physiologic variables of CF and COPD subjects in the FEV_1_% dynamic classes. Notched boxplots of age, AF product, BMI and slope of the least-squares trend line are shown for CF (6A-D) and COPD (6E-H) subjects in the FEV_1_% Dynamic Classes. Also shown is the distribution of the median difference between best FEV_1_% (CF subjects) and the MMRC dyspnea score (COPD subjects).

In the CF cohort, there were no differences in age, AF product, BMI, or slope of the least-squares trendlines between subjects in the three lung dynamic classes (Fig 6). Similarly, subjects in the BCCS FEV1 dynamic classes did not differ significantly in terms of age, AF product, BMI or slopes of the least-squares trendlines. The median difference from the best FEV_1_% is associated with a faster rate of lung function decline in CF patients [10]. Compared to CF patients with the stable phenotype, the hypervariable CF subjects had a higher median difference from the best FEV_1_% (HV/S ratio of 1.71 with a p value < .001) which is compatible with the higher risk associated with this hypervariable lung function phenotype.

### FEV_1_ dynamic classes vs clinical phenotypes

**Fig 7:**
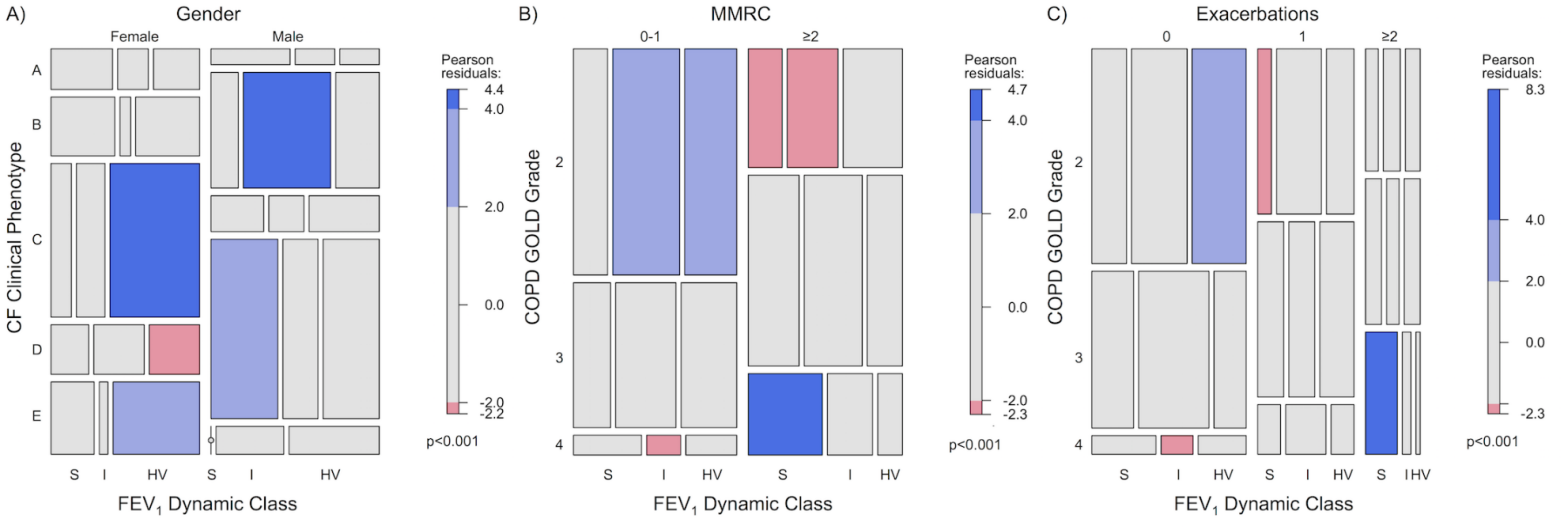
Mosaic plots of the FEV_1_% dynamic classes. Mosaic plots were used to assess positive or negative associations between clinical phenotypes and FEV_1_ dynamic classes with other categorical variables. Since width and length of the boxes are proportional to the observed frequencies in the datasets, the area of the boxes represent the proportion of subjects that share the attributes. To the extent that the observed proportions of subjects differs from expected proportions, the boxes are shaded to different levels of Pearson residuals. The associations between FEV_1_% Dynamic Class with MDCPs and gender in the CF cohort are shown (A). The associations between FEV_1_% Dynamic Class, COPD GOLD grades and either MMRC dyspnea scores or pulmonary exacerbations are depicted (B,C).

Mosaic plots were used to compare the distribution of categorical variables between the FEV_1_ dynamic classes (Fig 7). Mosaic plots assume proportional distribution of the categorical variables to be the same in the subclasses as it is in the whole population [22,23]. The size of the boxes is proportional to the number of subjects that share the attributes of the categories. Boxes that statistically deviate significantly from expected result are shaded.

In the CF cohort, the stable CF FEV_1_ dynamic class (S) occurred 2.1 times as often than expected (expected values calculated by the Chi-squared test of independence) in male subjects of the regional CF MDCP D, a milder clinical phenotype (Fig 7A). In contrast, the females in regional CF MDCP C were classified twice as much as expected with the hypervariable FEV_1_ dynamic class HV. This clinical phenotype is female-dominated and has a high rate of phenotype transition over a three-year period [13]. Finally another strong association noted in CF data is one between the FEV_1_ dynamic class I with male subjects in regional CF MDCP B (2.1 times the expected level), an older, male-dominated phenotype with poor lung function and nutritional status and a high rate of death and lung transplantation.

The COPD cohort demonstrated a strong positive association between the stable COPD FEV1 dynamic class S in GOLD grade 4 patients with the number of pulmonary exacerbations (4.0 times the expected value) and high MMRC dyspnea scores (2.4 times the expected value) (Fig 7B and 7C). In contrast, the more variable COPD FEV1 dynamic class I was associated with milder GOLD grade 2 and lower MMRC dyspnea scores (1.3 times expected value). In a similar manner, COPD FEV1 dynamic class HV was positively associated with milder GOLD grade 2 patients and fewer exacerbations (1.4 times the expected value).

## Discussion

We demonstrate the feasibility of modeling individual FEV_1_% with median regression splines in CF and COPD subjects. Variables capturing the dynamics of the splines and their predicted first and second derivatives were used to form lung function variability classes. A dimension reduction strategy that reduced the data complexity identified groups of subjects with similar spline dynamics. This strategy retained variables which capture the magnitude and variability of the changes in these curves and maintained classification error rates in the 5–10% range. In larger studies, it will likely be more useful to further refine the FEV_1_ dynamic classes by using a greater number of the variables for class formation.

The dynamics of FEV_1_ were not included in the formation of the regional CF phenotypes and yet the different airway lung function dynamic classes were enriched in specific clinical phenotypes. For example, males in the regional CF clinical phenotype, CF MDCP D, were positively associated with the most stable FEV_1_ dynamic class S consistent with this phenotype’s milder, more stable clinical course. The intermediate CF FEV_1_ dynamic class I was over-represented in CF MDCP B, another older, male-dominated phenotype that is associated with the highest rates of death and lung transplantation. Finally, the hypervariable CF lung function phenotype was seen more frequently than expected in two female-dominated CF clinical phenotypes, MDCP C and E. CF MDCP C is clinically unstable with the highest rate of phenotype transition over three years and a high frequency of sputum cultures positive for fungal species [13]. It is unclear if the distinctive characteristics of the airway microbiome in these two phenotypes are responsible for the lung function variability.

The modeled lung function in the COPD cohort behaved distinctly from the CF cohort. The ranges of the estimated second derivatives of the COPD cohort was approximately 10% of the CF cohort which likely resulted from differences in the pathophysiology of the disease *and* the indications for the spirometry assessments i.e. irregularly-timed, clinically indicated studies for the CF population versus more regularly-spaced research spirometries in the BCCS cohort. Although the COPD cohort showed no strong associations between gender, the FEV_1_ dynamic classes and GOLD 2,3,or 4 grades, it surprisingly showed positive associations between the stable COPD FEV_1_ dynamic class (S), the severe GOLD grade 4 and both the number of exacerbations and higher MMRC dyspnea scores. The more variable FEV_1_ dynamic classes (class I and HV) were enriched in subjects with milder GOLD grades 2 and 3 and lower pulmonary exacerbations rates and MMRC dyspnea scores. In this COPD cohort lung function variability appeared inversely associated with the MMRC scores, exacerbation rates and underlying FEV_1_%. A survivor effect might explain the overrepresentation of the stable FEV_1_ dynamic class in GOLD grade 4. With very low FEV_1_, even small changes in FEV1 may lead to exacerbation.

This analysis modeled individual lung function measurements over time, summarized the changes, and then used the summary statistics to generate groups of subjects with similar dynamic behavior. Depending on the modeling objective, there are many alternative statistical models that can be considered. Linear mixed models are a common approach for modeling longitudinal data, where population-level mean response is modeled as a linear function of time and subject specific random effects are used to characterize the between subject and within subject variations. Szczesniak et al. successfully extended this approach in CF patients by using semi-parametric mixed models and penalized regression splines for a more flexible mean structure in the mixed modeling approach [25]. Their modeling of FEV_1_% decline using semi-parametric nonlinear models suggested that the trends for survivors and non-survivors diverged around 12 years of age. Using a Bayesian framework, Moss et al. investigated several change point models including mixture models, for the decline of lung function in children and adolescents with CF [8]. The objective was to estimate the magnitude of lung function decline, along with the factors associated with the decline, and still account for individual changes over time. The results supported the hypothesis that there are two groups of CF adolescents, one with a change point in the decline of lung function and one without a change point. Recently, Szczesniak et al. used the statistical approach of sparse functional principal components analysis to classify patients into distinct phenotypes using longitudinal FEV1 trajectories [9]. The classes were determined by a quantile rule using the first and third tertiles of scores from the first functional principal component. This is in contrast to our study where the groups were determined by the data in an unsupervised method.

Two additional studies demonstrated the utility of characterizing individual variability of FEV_1_% in CF and COPD populations using a single measure of lung function variability [10,11]. Morgan et al. demonstrated that the median deviation from the best FEV_1_ was the best predictor of lung function loss over a two-year period. Casanova et al. looked at a very different model of individual FEV_1_ variability i.e. the number of spirometry studies that exceeded the annual loss of lung function and found that FEV_1_ variability was not significantly associated with 2 year mortality. In contrast, this current study captures the complexity of lung function dynamics using multiple measures of the variability of the median regression splines.

There are several limitations of this study. The generalizability of the clinical associations presented in this study will be limited to the extent that the datasets may not represent the adult CF population or the general COPD population. Comparisons of basic demographic data of the CF cohort and the CF Foundation Patient Registry and also the BCCS cohort to the broader ECLIPSE dataset suggest that the findings will generally hold up in larger studies [4,24]. Another limitation involves how the number and timing of the spirometry data affects spline dynamics or FEV_1_ dynamic class assignment in both clinical cohorts. We expect that stable patients with milder CF clinical phenotypes would have fewer studies and would more likely be classified into the stable FEV1 dynamic class. To the extent that CF dynamic class formation is based on real life use of spirometry, CF lung function class formation with encounter-based registry data may improve its associations with clinical phenotypes. In contrast, mapping COPD patients with clinically-indicated spirometry data into these COPD lung function dynamic classes would likely not provide valid insights.

In summary, nonlinear modeling of lung function using median regression splines provides unique opportunities to quantify short-term airway physiology variability and to associate this variability with important clinic parameters including clinical phenotypes and other dynamic factors that alter airway physiology such as the airway microbiome and metabolome or the host acute immune response.

## Supporting information

S1 Fig Median regression spline and derivative generation and dynamic variables. Each subject’s FEV_1_% is modeled and estimated using a median regression spline with the smoothing parameter chosen by generalized cross validation (GCV) based on the quantile function. The median smoothing spline is the solution to a minimization problem and fits a piecewise cubic polynomial with the join points at the unique set of time or *x*-values. The piecewise polynomials are constructed so that the entire curve has continuous first and second derivatives. The analysis and computations were performed using R and the qsreg median spline regression function with the default parameters [21,26].
*Case Report*. A case report demonstrates how the median regression spline was used to capture the short-term dynamics of the FEV_1_% predicted. The patient is a 43-year old, pancreatic sufficient woman with a CFTR genotype of dF508/Q372Q. The latter, synonymous mutation is a variation of a canonical splice site sequence at the exon-intron boundary at Exon 7. Although dF508 is considered not responsive to ivacaftor therapy, the responsiveness of this specific Q372Q mutation was unclear. The patient grows *Escherichia coli*, *Aspergillus fumigatus*, *Scedosporium azoospermia* and methicillin-sensitive *Staphylococcus aureus*. Therapies include inhaled 7% hypertonic saline, recombinant human DNase, and tobramycin. Oral medications include the chronic use of oral azithromycin and voriconazole. In the past, she was on chronic inhaled amphotericin because of recurrent hemoptysis.
The Figure demonstrates the summary variables of the median regression spline that were used to capture the dynamics of the FEV_1_ used in the CF and COPD cohorts. The measured FEV_1_% predicted values (black circles) and the median spline (black curve) are shown for the patient in the case report. Also depicted, are the periods of oral antibiotic (horizontal grey) and intravenous antibiotic (horizontal black) therapy. Off-label use of ivacaftor therapy is indicated with the horizontal red bar. The interval between the earliest and most recent FEV_1_ value was subdivided into 1000 equal spaced points. From these points, the median, as well as the 5^th^ and 95^th^ percentile values were predicted. Additional derived variables include the range between the values of 5^th^ and 95^th^ percentile points (vertical grey) bar and the number of local maxima and minima (red triangles). These variables were also identified in the first and second derivatives. Finally, in addition to these variables, the slope of the least squares regression line (thin grey trendline) and the effective degrees of freedom of the median regression spline were estimated.
(TIF)

S1 Table Multi-dimensional Clinical Phenotypes (MDCPs). The mean values of the FEV1% predicted (NHANES III), Brasfield chest Xray score, age, AF product, BMI and percent of male subjects in each of the regional Cystc Fibrosis clinical phenotypes. Adapted from PLOS ONE 2015;10:e0122705 (13).
(TIF)

S2 Table Cystic fibrosis cohort lung function. (XLS)

S3 Table Chronic obstructive pulmonary disease cohort lung function. (XLS)

## References

[1] Fletcher C, Peto R. The natural history of chronic airflow obstruction. Br Med J. 1977;1: 1645–1648. 871704 10.1136/bmj.1.6077.1645 PMC1607732

[2] Burrows B, Cline MG, Knudson RJ, Taussig LM, Lehowitz MD. A Descriptive Analysis of the Growth and Decline of the FVC and FEV1. Chest. 1983;83: 717–724. doi: 10.1378/chest.83.5.717 6839813 10.1378/chest.83.5.717

[3] Ware JH, Dockery DW, Louis TA, Xu X, Ferris BG, Speizer FE. Longitudinal and Cross-Sectional Estimates of Pulmonary Function Decline in Never-Smoking Adults. Am J Epidemiol. 1990;132: 685–700. 2403109 10.1093/oxfordjournals.aje.a115710

[4] Lange P, Celli B, Agustí A, Boje Jensen G, Divo M, Faner R, et al Lung-Function Trajectories Leading to Chronic Obstructive Pulmonary Disease. N Engl J Med. 2015;373: 111–122. doi: 10.1056/NEJMoa1411532 26154786 10.1056/NEJMoa1411532

[5] Schluchter MD, Konstan MW, Drumm ML, Yankaskas JR, Knowles MR. Classifying Severity of Cystic Fibrosis Lung Disease Using Longitudinal Pulmonary Function Data. Am J Respir Crit Care Med. 2006;174: 780–786. doi: 10.1164/rccm.200512-1919OC 16858011 10.1164/rccm.200512-1919OC PMC2648062

[6] Exuzides A, Colby C, Briggs A, Lomas DA, van-Mölken MR, Tabberer M, et al Statistical Modeling of Disease Progression for Chronic Obstructive Pulmonary Disease Using Data from the ECLIPSE Study. Med Decis Making. 2015; 0272989X15610781. doi: 10.1177/0272989X15610781 26449490 10.1177/0272989X15610781

[7] Konstan MW, Wagener JS, VanDevanter DR, Pasta DJ, Yegin A, Rasouliyan L, et al Risk factors for rate of decline in FEV1 in adults with cystic fibrosis. J Cyst Fibros. 2012;11: 405–411. doi: 10.1016/j.jcf.2012.03.009 22561369 10.1016/j.jcf.2012.03.009 PMC4086189

[8] Moss A, Juarez-Colunga E, Nathoo F, Wagner B, Sagel S. A comparison of change point models with application to longitudinal lung function measurements in children with cystic fibrosis. Stat Med. 2016;35: 2058–2073. doi: 10.1002/sim.6845 27118629 10.1002/sim.6845

[9] Szczesniak RD, Li D, Su W, Brokamp C, Pestian J, Seid M, et al Phenotypes of Rapid Cystic Fibrosis Lung Disease Progression during Adolescence and Young Adulthood. Am J Respir Crit Care Med. 2017; doi: 10.1164/rccm.201612-2574OC 28410569 10.1164/rccm.201612-2574OC PMC5564675

[10] Morgan WJ, VanDevanter DR, Pasta DJ, Foreman AJ, Wagener JS, Konstan MW, et al Forced Expiratory Volume in 1 Second Variability Helps Identify Patients with Cystic Fibrosis at Risk of Greater Loss of Lung Function. J Pediatr. 2016;169: 116–121.e2. doi: 10.1016/j.jpeds.2015.08.042 26388208 10.1016/j.jpeds.2015.08.042

[11] Casanova C, Aguirre-Jaíme A, de Torres JP, Pinto-Plata V, Baz R, Marin JM, et al Longitudinal assessment in COPD patients: multidimensional variability and outcomes. Eur Respir J. 2014;43: 745–753. doi: 10.1183/09031936.00096913 24072210 10.1183/09031936.00096913

[12] Hankinson JL, Odencrantz JR, Fedan KB. Spirometric Reference Values from a Sample of the General U.S. Population. Am J Respir Crit Care Med. 1999;159: 179–187. doi: 10.1164/ajrccm.159.1.9712108 9872837 10.1164/ajrccm.159.1.9712108

[13] Conrad DJ, Bailey BA. Multidimensional Clinical Phenotyping of an Adult Cystic Fibrosis Patient Population. PLOS ONE. 2015;10: e0122705 doi: 10.1371/journal.pone.0122705 25822311 10.1371/journal.pone.0122705 PMC4378917

[14] Eagan TML, Ueland T, Wagner PD, Hardie JA, Mollnes TE, Damås JK, et al Systemic inflammatory markers in COPD: results from the Bergen COPD Cohort Study. Eur Respir J. 2010;35: 540–548. doi: 10.1183/09031936.00088209 19643942 10.1183/09031936.00088209

[15] Aarli BB, Calverley PMA, Jensen RL, Eagan TML, Bakke PS, Hardie JA. Variability of within-breath reactance in COPD patients and its association with dyspnoea. Eur Respir J. 2015;45: 625–634. doi: 10.1183/09031936.00051214 25359342 10.1183/09031936.00051214

[16] Mahler DA, Wells CK. Evaluation of Clinical Methods for Rating Dyspnea. Chest. 1988;93: 580–586. doi: 10.1378/chest.93.3.580 3342669 10.1378/chest.93.3.580

[17] Quanjer PH, Tammeling GJ, Cotes JE, Pedersen OF, Peslin R, Yernault JC. Lung volumes and forced ventilatory flows. Report Working Party Standardization of Lung Function Tests, European Community for Steel and Coal. Official Statement of the European Respiratory Society. Eur Respir J Suppl. 1993;16: 5–40. 8499054

[18] Murrell P. R Graphics, CRC Computer Science & Data Analysis. Chapman & Hall; 2005.

[19] Breiman L. Random Forests. Mach Learn. 2001;45: 5–32.

[20] Maechler M, Rousseeuw P, Struyf A, Hubert M, Hornik K. cluster: Cluster Analysis Basics and Extensions [Internet]. 2015. http://CRAN.R-project.org/package=cluster

[21] R Core Team. R: A language and environment for statistical computing. R Foundation for Statistical Computing. [Internet]. Vienna, Austria.; 2014. <http://www.R-project.org/>

[22] Friendly M. Mosaic Displays for Multi-Way Contingency Tables. J Am Stat Assoc. 1994;89: 190–200. doi: 10.1080/01621459.1994.10476460

[23] Hornik K, Zeileis A, Meyer D. The Strucplot Framework: Visualizing Multi-way Contingency Tables with vcd. J Stat Softw. 2006;17: 1–48.

[24] Cystic Fibrosis Foundation Patient Registry. Cystic Fibrosis Foundation; 2014.

[25] Szczesniak RD, McPhail GL, Duan LL, Macaluso M, Amin RS, Clancy JP. A semiparametric approach to estimate rapid lung function decline in cystic fibrosis. Ann Epidemiol. 2013;23: 771–777. doi: 10.1016/j.annepidem.2013.08.009 24103586 10.1016/j.annepidem.2013.08.009

[26] Nychka D, Furrer R, Sain S. fields: Tools for Spatial Data. R package version 8.2–1 [Internet]. 2015. <http://CRAN.R-project.org/package=fields>

