# Supplementary figures and images for: Median regression spline modeling of longitudinal FEV1 measurements in cystic fibrosis (CF) and chronic obstructive pulmonary disease (COPD) patients
Source: PLoS One. 2017 Dec 20;12(12):e0190061. doi: 10.1371/journal.pone.0190061 (PMC5738083; doi:10.1371/journal.pone.0190061)

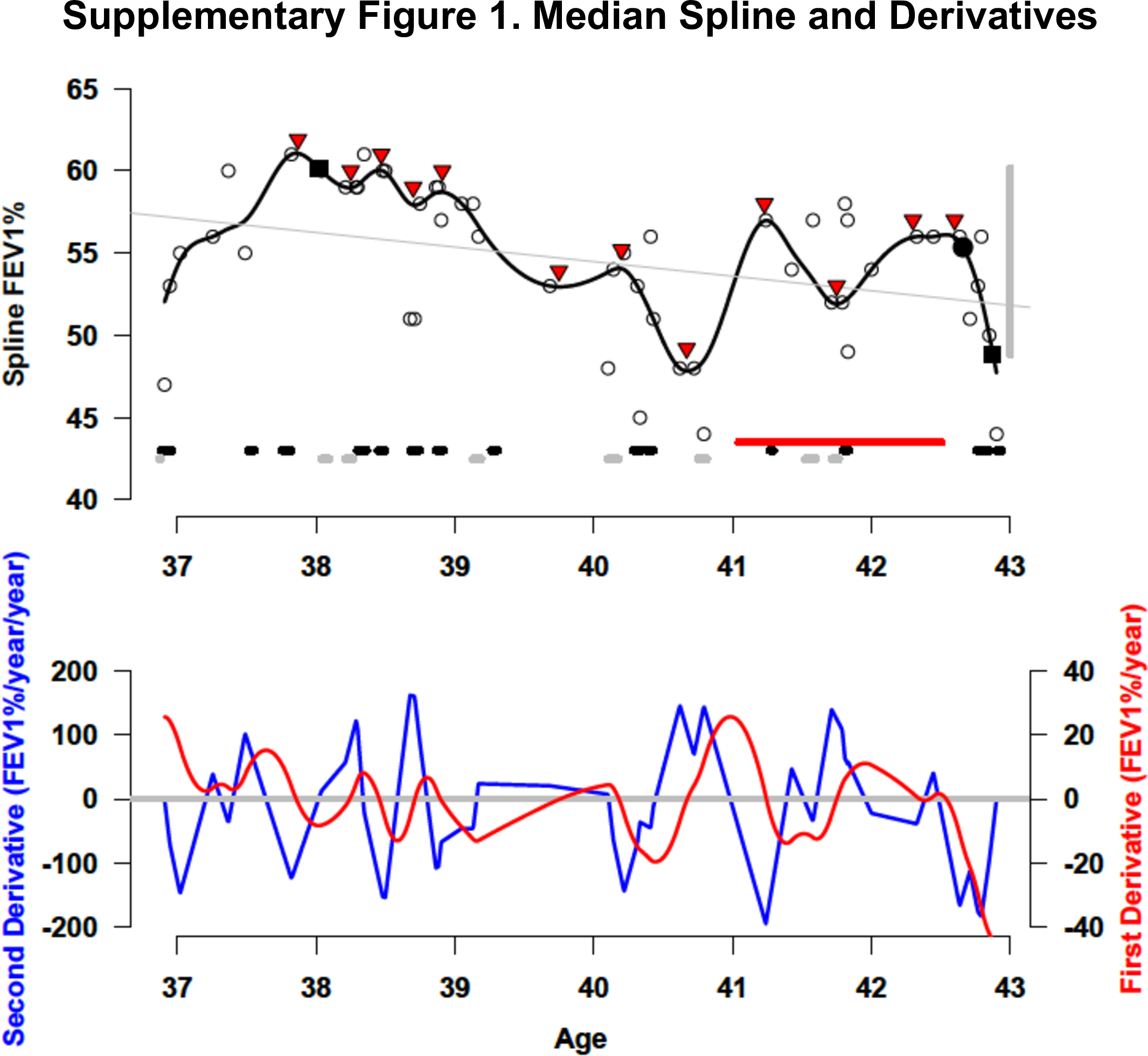

Supplement: S1 Fig — Each subject’s FEV1% is modeled and estimated using a median regression spline with the smoothing parameter chosen by generalized cross validation (GCV) based on the quantile function. The median smoothing spline is the solution to a minimization problem and fits a piecewise cubic polynomial with the join points at the unique set of time or x-values. The piecewise polynomials are constructed so that the entire curve has continuous first and second derivatives. The analysis and computations were performed using R and the qsreg median spline regression function with the default parameters [21,26]. Case Report. A case report demonstrates how the median regression spline was used to capture the short-term dynamics of the FEV1% predicted. The patient is a 43-year old, pancreatic sufficient woman with a CFTR genotype of dF508/Q372Q. The latter, synonymous mutation is a variation of a canonical splice site sequence at the exon-intron boundary at Exon 7. Although dF508 is considered not responsive to ivacaftor therapy, the responsiveness of this specific Q372Q mutation was unclear. The patient grows Escherichia coli, Aspergillus fumigatus, Scedosporium azoospermia and methicillin-sensitive Staphylococcus aureus. Therapies include inhaled 7% hypertonic saline, recombinant human DNase, and tobramycin. Oral medications include the chronic use of oral azithromycin and voriconazole. In the past, she was on chronic inhaled amphotericin because of recurrent hemoptysis. The Figure demonstrates the summary variables of the median regression spline that were used to capture the dynamics of the FEV1 used in the CF and COPD cohorts. The measured FEV1% predicted values (black circles) and the median spline (black curve) are shown for the patient in the case report. Also depicted, are the periods of oral antibiotic (horizontal grey) and intravenous antibiotic (horizontal black) therapy. Off-label use of ivacaftor therapy is indicated with the horizontal red bar. The interval between the [file pone.0190061.s001.tif]

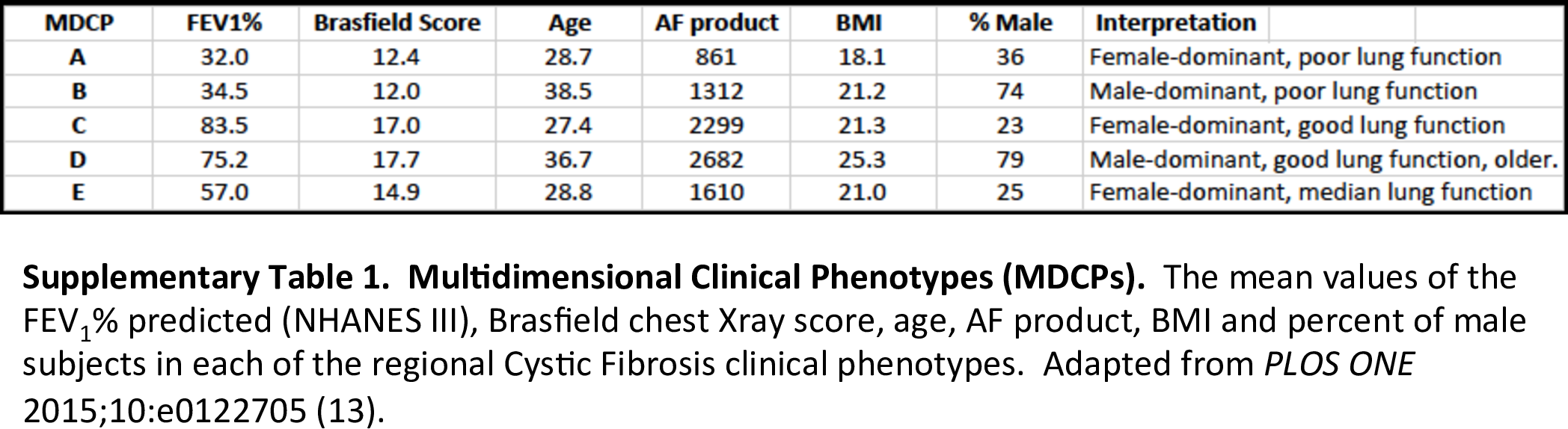

Supplement: S1 Table — The mean values of the FEV1% predicted (NHANES III), Brasfield chest Xray score, age, AF product, BMI and percent of male subjects in each of the regional Cystc Fibrosis clinical phenotypes. Adapted from PLOS ONE 2015;10:e0122705 (13). (TIF) [file pone.0190061.s002.tif]
